# Mr. Wilson, on the Medical Library

**Published:** 1803-10-01

**Authors:** John Wilson

**Affiliations:** Gillingham


### *To the Editors of the Medical and Physical Journal.*

GENTLEMEN,

YOUR strict attention to whatever can promote the interests of our valuable profession demands the thanks of the medical world, and emboldens me to presume that the following remarks will meet your approbation.

The

The enormous price of books is an evil calling loudly for redress; it is a subject too interesting to the literary community to be longer neglected. The fashion of publishing every production in a splendid style, is a serious obstacle to the diffusion of knowledge. Is it vanity in the author, or folly in the public, that gives us such fine editions in quarto and folio? Every prospectus of publication in these times, is more prolix in its detail of fine wove paper and handsome type, than explanatory of the merits of the work. In the wide range of letters, from the Principia of Newton to the history of Jack the Giant Killer, we have such emphatic description of the quality of paper and type, that an ignorant person might be led to believe all human learning was manufactured at the mill and foundery. Probably the citizen supposed the bookbinder was a profound scholar, when in fitting up his villa he expressed a particular desire that his library might be composed of books *elegantly bound*, without any anxiety respecting their contents. Sir William Blackstone honours the Faculty, when recommending the study of the law, "to complete the character of general and extensive knowledge; a character which their profession, beyond all others, has remarkably deserved."* I fear this remark will soon be applicable to a *fortunate few*, if the price of learning continues to advance.

Truth and science, Gentlemen, are like Lavinia, "when unadorned, adorned the most;" and it becomes the guardians of literature to protect their charge from the dangerous innovations of frivolous fashion or vain parade. The evils arising out of this ridiculous propensity to gaudy shew are not confined merely to the pocket, but must have a farther and equally pernicious effect. Is it not to be feared that the exterior will subtract from our attention to the intrinsic value of the book; and that in our admiration of art we forget the utility of science? Can any decorations add to the importance of the works of Hypocrates, Boerhaave, Cullen, Monro, Hunter, and Darwin? Certainly not. These authors may be ranked the first in the scale of medical and physiological repute; their writings have been numerous; but to enable the professional world to profit by their labours, they should be published in the cheapest forms possible.

For

* Vide Vol. I. p. 13. Commentaries on the Laws of England.

For the honour of science, let her be clothed in plain attire, and consign the trash of the day to the livery of red Morocco and gold leaf. The nobleman of high rank and acknowledged worth is contented on common occasions with a respectable decency of exterior, whilst the fluttering coxcomb eclipses the rainbow. Novels, romances, and plays, are on a parallel with the gaudy Fribble; and as they possess little internal worth, I think their readers may properly be taxed for their folly. But in a grave, studious profession, like ours, embracing in its perfection *all* the sciences, wherein it is justly said, Ars longa et vita brevis, the means of information cannot be too much facilitated; nor can I sufficiently lament the melancholy effects that I hourly feel of this universal mania.

I know it may be said, that the high price of books arises partly from the heavy duties on paper. Admitted, but why add to that evil by unnecessary, extraneous, and expensive embellishments? May we not reasonably suppose that government saw our passion for beauty of art, and resolved to share the spoil with the artist? If philosophers had not been seduced by fashion or vanity, this folly would have been confined to foolish works alone.

The genius and industry of the present age have triumphed over the obstacles arising out of expence, but surely it is a legitimate inference that the profound discoveries and elegant improvements in every branch of medical science would have been more amply diffused, if the poor in pocket could have attained the possession of them.

Every author in medicine and surgery should reflect that there is a particular description of the Faculty precluded from the means of improvement by the enormous price of scientific books. It is in the metropolis, and large towns only, where professional men can obtain literary enjoyment at an easy rate, by forming themselves into societies; but what is to become of the country practitioner, who within his circle is an equally important, and ought to be an equally learned and respectable character? The objects he has to practice on, hold life and health in as high estimation as the pampered inhabitants of wealthy cities, and if public considerations might be indulged in such a case, we might perhaps in truth accord with Dr. Adam Smith,* that

* See his incomparable Inquiry into the Nature and Causes of the Wealth of Nations.

that they more immediately form the basis of national opulence. If a solitary newspaper, penetrating his obscure retreat, announces to him the publication of a valuable work, he longs to peruse it; but the fine contour of the type, the beautiful texture of the paper, and probably the elegant binding of the splendid quarto, form so many insuperable obstacles to his *borrowing* it, how then should he *buy* it? he can only sigh over the sum total of its nominal value. The Zoonomia of Dr. Darwin (a work that may vie with any, whether antient or modern) was first published in a form that limited its distribution, and consequently its utility; the same may be said of Mr. John Bell's Principles of Surgery; its enormous price places it beyond the attainment of men in moderate practice, and these are the men who must require the result of another's experience to supply the deficiencies of their own. I am not ashamed to acknowledge, Gentlemen, that I *feel*; yes, I sensibly *suffer*, from the evils of which I presume to complain. My little library might have been twice its magnitude, if half of its prime value had not been sunk in the quality of type and paper. I am unable to store my mind with the estimable discoveries of my cotemporaries, because I could barely afford to purchase truth in her naked simplicity, and my ambition will never extend to the possession of her decked in *imperial* attire.

It is a melancholy consideration, that whilst almost every other class of men are in a state of improvement, the Faculty are certainly going retrogade; I speak to their revenue; competition has reduced the Profession to the lowest ebb, consistent with comfortable support. Half a century has produced an enormous advance in the price of all the articles of the materia medica, as well as of the necessaries of life; but no equivalent advance of charge by the apothecary has taken place. In short, such is the state of the country practitioner, that empiricism must soon supercede science, if every facility is not afforded him to procure information.

To remedy these evils, I humbly beg leave to recommend that all writers on subjects connected with medicine and surgery should publish on coarse paper with a plain type; it may be profitable too to intrude a little more on the margin. Dr. Darwin suggested the propriety of making the letter-press a spectrum with the ground, or paper, and perhaps by its superior distinctness we should be able to use paper of a quality still less expensive. The literary works of our continental neighbours, are almost all of them published on a coarse paper, and perhaps the general diffusion of knowledge over France and Germany, in spite of their political slavery, may be attributed to the easy means of attainment.

Consistently with the above views, I call upon you, Gentlemen, to give the first example of departure from fashion. It would be highly honourable, and, I judge, equally profitable, to stand forward the first instance of independant character; add to this, that the circulation of your work would be infinitely extended, and the discoveries in medicine and surgery more widely promulgated. If I did not conceive the Medical and Physical Journal to be a miscellany of deserved repute, I should have presented my remarks to the Editors of some other publication; and assure you, that I do not compliment when I say, that by your giving an example of literary economy, you will influence authors as much as you will benefit readers. The Faculty, Gentlemen, have a sensible interest in my appeal to your prudence, and I presume will not refuse to join in my request, that you will abstract a little from the beauty of your work to render it of more universal utility.

It is possible some may object that any change in your mode of publication will diminish its value by destroying its uniformity; and that the taste of the public must be complied with. But I am sure, that however the country practitioner may hunger for knowledge, he cannot at present taste it; and it is of very little importance to a man anxious in the pursuit of professional information, whether truth be depicted on brown paper or vellum.

To shew that a scientific publication can sustain its character without condescending to put on a fashionable dress, I refer you to the Gentleman's Magazine, which, I believe, is not considered less valuable, because it is less *pretty* than the ephemeral productions of many moderns, and I think I dare venture to predict, that the Medical and Physical Journal will not sink in the public esteem, whilst conducted on its present principles, however plain the paper and type may be.

I hope, Gentlemen, to be believed when I declare myself a sincere admirer of the works of art; but when I see learning reverenced the more for its trappings, I fear a decay of public wisdom. The art of printing was invented to communicate knowledge, and has been the happy means of diffusing sciences and arts, improving our morals and politics, and extending human civilization through the remotest regions; but it will cease to be subservient to such purposes, purposes, because it bids fair to become the *primary* object of consideration.

In offering you the above remarks, I hope, Gentlemen, I am not actuated entirely by a selfish motive, and I beg that they who may see no danger, or who are too rich to feel any inconvenience, from the subject of my complaint, will give me credit for a small share of anxiety respecting the welfare of that Profession which I am desirous not to disgrace; but if my poverty of pocket is deemed criminal, I must stand convicted, and assure you that if knowledge is to be sold by *aspect* instead of by sterling worth, my head will soon be as light as my purse; I shall be reduced to the distress of the mortified connoisseur, for I cannot purchase the picture on account of the expence of the frame.

I am, &c.

*Gillingham, Aug.* 9, 1803. JOHN WILSON.

